# The Effect of Environmental pH during *Trichothecium roseum* (Pers.:Fr.) Link Inoculation of Apple Fruits on the Host Differential Reactive Oxygen Species Metabolism

**DOI:** 10.3390/antiox10050692

**Published:** 2021-04-28

**Authors:** Zhanhong Han, Zhenyu Wang, Yang Bi, Yuanyuan Zong, Di Gong, Bin Wang, Baojun Li, Edward Sionov, Dov Prusky

**Affiliations:** 1 College of Food Science and Engineering, Gansu Agricultural University, Lanzhou 730070, China; hanzhanhong009@163.com (Z.H.); 18709493393@163.com (Z.W.); gongdi531@163.com (D.G.); wangbin_1519@163.com (B.W.); libaojun_farm@163.com (B.L.); edwardsio@volcani.agri.gov.il (E.S.); dovprusk@volcani.agri.gov.il (D.P.); 2 Department of Postharvest Science of Fresh Produce, Agricultural Research Organization, Rishon LeZion 7505101, Israel

**Keywords:** *Trichothecium roseum*, environmental pH, reactive oxygen species, ascorbate-glutathione cycle

## Abstract

*Trichothecium roseum* is an important postharvest pathogen, belonging to an alkalizing group of pathogens secreting ammonia during fungal growth and colonization of apple fruits. Fungal pH modulation is usually considered a factor for improving fungal gene expression, contributing to its pathogenicity. However, the effects of inoculation with *T. roseum* spore suspensions at increasing pH levels from pH 3 up to pH 7, on the reactive oxygen species (ROS) production and scavenging capability of the apple fruits, affecting host susceptibility, indicate that the pH regulation by the pathogens also affects host response and may contribute to colonization. The present results indicate that the inoculation of *T. roseum* spores at pH 3 caused the lowest cell membrane permeability, and reduced malondialdehyde content, NADPH oxidases activity, O_2_^●−^ and H_2_O_2_ production in the colonized fruit. Observations of the colonized area on the 9th day after inoculation at pH 3, showed that the rate of O_2_^●−^ production and H_2_O_2_ content was reduced by 57% and 25%, compared to their activities at pH 7. In contrast, antioxidative activities of superoxide dismutase, catalase and peroxidases of fruit tissue inoculated with spores’ suspension in the presence of a solution at pH 3.0 showed their highest activity. The catalase and peroxidases activities in the colonized tissue at pH 3 were higher by almost 58% and 55.9%, respectively, on the 6th day after inoculation compared to inoculation at pH 7. The activities of key enzymes of the ascorbate-glutathione (AsA-GSH) cycle and their substrates and products by the 9th day after fruit inoculation at pH 3 showed 150%, 31%, 16%, and 110% higher activities of ascorbate peroxidase, monodehydroascorbate reductase, dehydroascorbate reductase and glutathione reductase, respectively, compared to pH 7. A similar pattern of response was also observed in the accumulation of ascorbic acid and dehydroascorbate which showed a higher accumulation at pH 3 compared to the colonization at pH 7. The present results indicate that the metabolic regulation of the pH environment by the *T. roseum* not only modulates the fungal pathogenicity factors reported before, but it induces metabolic host changes contributing both together to fungal colonization.

## 1. Introduction

Pathogens can dynamically alter the local pH at an infection site to suit the increasing expression of pathogenicity factors and the enzymatic arsenal that contribute to pathogenicity [1]. The ability to modify pH may be expressed in either direction, and fungi that raise or reduce it are described as ‘alkalizing fungi’ or ‘acidifying fungi’, respectively. Ambient alkalization by fungi is achieved by active secretion of ammonia, which is produced as a result of protease activity and deamination of amino acids. Other postharvest pathogens, such as *Penicillium expansum*, *Penicillium digitatum*, *Penicillium italicum* [2], *Botrytis cinerea* [3], and *Sclerotinia sclerotiorum* [4,5], utilize tissue acidification by organic acids to support their attack. *Trichothecium roseum* is an important postharvest pathogen with a wide range of fruit hosts [6] such as apples, pears, muskmelons, apricots, tomatoes, and other fruit [6]. It was recently reported to belong to the alkalinizing fungus group, which can activate various extracellular enzymes in lesions in a neutral or slightly alkaline environment [7].

pH modulation is considered a main mechanism for differentially activating pathogenicity factors in the pathogen but the effect of the modulation of pH by the fungus on the host response has not been reported. Reactive oxygen species (ROS) is an important pathogenicity factor of pathogens [8]. Overproduction of ROS in the host causes lipid peroxidation and damage of the cell membrane in the early stages of infection, leading to the reduction of host disease resistance [8]. Bao et al. (2014) found that *Fusarium sulphureum* with stronger pathogenicity not only led to higher ROS accumulation in potato tubers, but also weakened the ROS scavenging ability by inhibiting the activities of antioxidant enzymes and the ascorbate-glutathione (AsA-GSH) cycle [9]. Furthermore, *Botrytis cinerea* was able to enhance its pathogenicity in tomato leaves by decreasing the enzyme activities of the AsA-GSH cycle, suggesting the importance of the antioxidant mechanism regulation during fungal pathogenicity [10].

Studies have shown that environmental pH can regulate the production and scavenging of ROS in plants. When bean cells were transferred from low pH (5) to pH 7.2 buffer medium, the activities of NADPH oxidases, resulted in a rapid production of H_2_O_2_ [11]. In addition, in a condition of pH 4.0 an increasing activity of the AsA-GSH cycle, reduction of H_2_O_2_ content, and inhibition of membrane lipid peroxidation was observed in ginger seedlings [12]. In conditions where muskmelon tissue was treated with oxalic acid at pH 1.8, and increased the activity of the ASA-GSH cycle, this induced the expression of *CmPOD*, inhibited O_2_^●−^ and H_2_O_2_ accumulation, and a delayed membrane lipid peroxidation, and increased cell membrane integrity [13]. These studies also showed that ammonia altered the K^+^/H^+^ concentration of intracellular and extracellular cells and promoted Ca^2+^ influx [14]. Ethylene glycol-bis-(b-amino-ethyl ether) N, N, N′, N′-tetraacetic acid (EGTA) treatment reduced the intracellular Ca^2+^ concentration of potato tubers and inhibited the expression of plasma membrane NADPH oxidases and NADPH oxidases activity, thereby reducing the production of ROS, which could be restored by adding exogenous Ca^2+^ [15]. In another host pathogen system *Colletotrichum coccodes* increased the pH of wounded tomato fruit by secreting ammonia, resulting in the activation of NADPH oxidases activity, and the induction of H_2_O_2_ synthesis, thereby resulting in an increase of its pathogenicity [16].

Although it has been reported that pathogens could increase the ROS level of the host by the mechanism of pH regulation and secretion of ammonia, the direct effect of the environmental pH during the inoculation of *T. roseum* on the host and metabolic effect on ROS production and scavenging in apple fruit have not been analyzed. In this study, ‘Fuji’ apples were inoculated with *T. roseum* spore suspension at different pH levels. The cell membrane permeability, malondialdehyde content, ROS production, scavenging enzymes activities, key enzymes activity, and substrate and product contents in the AsA-GSH cycle were analyzed to determine the effects of different levels of pH on antioxidant capacity in colonized apples. This analysis suggests that the pathogens enhance pathogenicity by modulating the environmental pH, transcriptionally activating fungal pathogenicity factors but also modulate the host metabolic response.

## 2. Materials and Methods

### 2.1. Fruit and Strain

Apples (*Malus domestica* Borkh. cv. Fuji) were harvested from a commercial orchard in Jingtai (37°38′ N, 105°34′ E, 1671 m altitude), Gansu Province, China. The type of soil is sandy, annual average temperature is 3.1–9.1 °C, and annual rainfall is 160–450 mm. The fruits were obtained from 15-year-old trees, planted in a 5 × 5 m plantation. The cultivation was in accordance with the local cultivation standard. The fruits were neat in appearance, of uniform size, and free from pests, disease, and mechanical injury, withTotal Soluble Solids values of 14.2 (TSS). Fruits were put into corrugated paper packing boxes after each fruit was netted. The packed fruits were transported to the Postharvest Biology and Technology Laboratory at Gansu Agricultural University on the day of harvest, stored in the cold room (4 ± 2 °C, with a relative humidity of 55%–60%), and were treated two days later.

*T. roseum* was isolated from the core of rotten apples and cultured on potato dextrose agar (PDA) medium.

### 2.2. Spore Suspension with Different pH Values

Spore suspensions were prepared according to the method of Eshel et al. [17]. Three dishes of *T. roseum* were cultured at 28 °C for 5 days, then 10 mL of disodium hydrogen phosphate-citric acid buffer at pH 3, pH 5, and pH 7 were added to each dish. Spores on the surface of the culture medium were gently scraped off with a sterile glass rod, filtered through four layers of sterile gauze, and the concentration of the spore suspension was counted with a hemocytometer and diluted to a concentration of 1 × 10^6^ spores/mL with sterile distilled water.

### 2.3. Fruit Inoculation

The fruits were inoculated according to the method of Gong et al. [18]. Apples were rewarmed for 24 h after removal from the cold room. After washing with tap water, they were surface-sterilized in 1% NaClO_3_ for 3 min. Then the apples were dried at room temperature, and four inoculation holes of 3 mm depth and 3 mm diameter were punched in the equator of the fruit with a sterilized iron nail. Ten microliters of spore suspensions of different pH were placed into the holes. After being dried, the fruits were placed into polyethylene bags and stored at room temperature (22 ± 2 °C, RH 85%–90%).

### 2.4. Maintenance of pH Value in Inoculation Site

A sterile disodium hydrogen phosphate-citrate buffer solution at pH 3, pH 5, and pH 7 was injected into the inoculation wells at 12 h intervals from the beginning of inoculation until the end of sampling time [17].

### 2.5. Sampling

The samples were collected according to the method described by Wang et al. [7]. At different days after inoculation, the peel and pulp of the diseased and healthy tissue (approximately 3 mm) at the leading edge junction were sampled, within 3 mm of the disease-health junction were taken, immediately frozen by liquid nitrogen, then stored at −80 °C until analyzed.

### 2.6. Determination of Cell Membrane Permeability and Malondialdehyde (MDA) Content

The cell membrane permeability was determined according to the method of Lester et al. [19] with minor modifications. Ten grams of samples were incubated in 40 mL of deionized water, and the conductivity was determined at 0 h and 3 h of incubation with a conductivity meter (DDS-307A; RIDAO, Shanghai) at 25 °C and recorded as E0 and E1, respectively. After that the samples were incubated in boiling water for 0.5 h, and then quickly cooled to determine the conductivity which was recorded as E2. The cell membrane permeability was calculated using the following formula:

Cell membrane permeability (%) = (E1 − E0)/ E2 × 100%

The MDA content was determined according to the method of Hodges et al. [20] with slight modifications. Frozen tissue (3 g) was homogenized with 6 mL precooled trichloroacetic acid (TCA) extract solution, and then centrifuged for 20 min at 12 000× *g*, at 4 °C. The absorbance of the reaction of 2 mL supernatant with 2 mL 0.67% (*w/v*) 2-thiobarbituric acid (TBA) was determined at 450 nm, 532 nm, and 600 nm after incubating in boiling water for 20 min. The MDA content was calculated according to the formula and expressed as nmol/g FW.

C_MDA_ (μmol/L) = 6.45 × (OD_532_ − OD_600_) − 0.56 × OD_450_

MDA content (nmol/g FW) = C_MDA_ (μmol/L) × Extraction volume (mL)/Fruit fresh weight (g)

### 2.7. Determination of NADPH Oxidase and Superoxide Dismutase (SOD) Activity

The NADPH oxidase activity was determined according to the method of Morré et al. [21] with slight modifications. Frozen tissue (3 g) was homogenized with 5 mL extract solution, and centrifuged at 4 °C and 7500× *g* for 15 min. The reaction mixture for NADPH oxidase activity contained 2 mL 50 mM Tris-HCl buffer (pH 7.5), 0.5 mM sodium 3,3′-[1-(Benzyl)-3,4-tetrazolium]-bis(4-methoxy-6-nitro) benzenesulfonate (XTT), 100 µM NADPH, and 200 µL microcapsule of cell membrane. The activity of NADPH oxidase was expressed as U g^−1^ FW, where U was defined as an increasing of 0.01 in absorbance per minute at 470 nm.

The SOD activity was determined according to the method of Supapvanich et al. [22] with slight modifications. Frozen tissue (3 g) was homogenized with 5 mL 50 mM phosphate buffer (pH 7.8). The reaction system contained 1.7 mL phosphate buffered saline (PBS) solution (pH 7.8, 50 mM), 300 μL 130 mM methionine, 300 μL 750 μM nitro blue tetrazolium (NBT), 0.3 mL 100 μM EDTA-Na_2_, 0.1 mL crude enzyme solution, and 100 μL riboflavin. The absorbance was measured at 560 nm. 50% inhibition of photoreduction of NBT and expressed as U g^−1^ FW.

### 2.8. Determination of Rate of O_2_^●−^ Production and H_2_O_2_ Content

The rate of O_2_^●−^ production was determined according to the method of Luo et al. [23] with some modifications. Frozen tissue (3 g) was homogenized with 5 mL 100 mM phosphate buffer (0.1% PVPP, pH 7.8) and centrifuged at 4 °C and 12,000× *g* for 20 min. Then 1 mL of supernatant was mixed with 1 mL 50 mM phosphate buffer (pH 7.8) and 1 mL 1 mM hydroxylamine hydrochloride, and incubated for 1 h at 25°C. Then 1 mL 17 mM *p*-aminobenzenesulfonic acid and 1 mL 7 mM α-naphthylamine were added and incubated at 25 °C for 20 min. The absorbance was measured at 530 nm. The O_2_^●−^ production rate was expressed as nmol/min/g FW.

The H_2_O_2_ content was determined using a kit (Nanjing Jiancheng Biotechnology Co., Ltd., Nanjing, China). Frozen tissue (3 g) was homogenized with 5 mL 1 mM phosphate buffer (pH 7). The reagents were added according to the instructions of the kit for H_2_O_2_ content determination. Absorbance was measured at 405 nm. The content of H_2_O_2_ was expressed as μmol g^−1^ FW.

### 2.9. Determination of Catalase (CAT) and Peroxidases (POD) Activities

The CAT activity was measured according to Fan et al. [24]. Frozen tissue (3 g) was homogenized with 5 mL 0.1 M sodium phosphate buffer. The homogenate was then centrifuged at 4 °C and 12,000× *g* for 20 min. The reaction mixture for CAT activity contained 50 mM phosphate buffer (pH 7.5), 20 mM H_2_O_2_, and 100 μL of crude enzyme. The activity of CAT was expressed as U g^−1^ FW, where U was defined as an increase of 0.01 in absorbance per minute at 240 nm.

POD activity was measured according to Venisse et al. [25]. Frozen tissue (3 g) was homogenized with 5 mL of 0.1 M acetic acid-sodium acetate buffer (containing 1% TritonX-100, 4% PVPP, and 1 mM PEG, pH 5.5). The homogenate was then centrifuged at 4 °C and 12,000× *g* for 20 min. The reaction mixture for POD contained 0.5 mL of 25 mM guaiacol, 200 μL of 30% H_2_O_2_, and 500 μL of crude enzyme extract. The activity of POD was expressed as U g^−1^ FW, where U was defined as an increase of 0.01 in absorbance per minute at 470 nm.

### 2.10. Determination of Key Enzyme Activities of the AsA-GSH Cycle

Crude enzyme solution was extracted according to the method described by Nakano and Asada [26]. Frozen tissue (3 g) was homogenized with 3 mL of pH 7.5 100 mM phosphate buffer (1 mM EDTA), and then centrifuged at 4 °C and 12 000× *g* for 30 min.

Ascorbate peroxidase activity was measured according to the method reported by Nakano and Asada [26]. The reaction system contained 2 mL 100 mM phosphate buffer (pH 7.5, containing 1 mM EDTA), 0.8 mL 3 mM ascorbic acid, 200 μL crude enzyme solution, and 0.5 mL 0.5 mM H_2_O_2_. The absorbance of the reaction system at 290 nm was recorded from 15 s after start-up for 2 min. The activity of APX was expressed as U g^−1^ FW, where U was defined as an increase of 0.01 in absorbance per minute at 290 nm.

Monodehydroascorbate reductase activity was measured according to the method reported by Nakano and Asada [26]. The reaction system was 2 mL 40 mM phosphate buffer solution (pH 8.0), 0.2 mL 10 mM sodium ascorbate, 0.1 mL 40 μM copper sulphate solution, 0.5 mL crude enzyme solution, and 0.2 mL of 0.2 mM NADPH. The absorbance at 340 nm was measured immediately after mixing and recorded for 2 min. The activity of DHAR was expressed as U g^−1^ FW, where U was defined as an increase of 0.01 in absorbance per minute at 340 nm.

Dehydroascorbate reductase activity was measured according to the method reported by Nakano and Asada. [26]. The reaction system was 100 μL crude enzyme solution, 300 μL 0.1 mM EDTA-Na_2_, 400 μL 2 mM GSH, 400 μL 0.5 mM DHA, and 2 mL 40 mM phosphate buffer solution (pH 8.0). The absorbance at 290 nm was measured immediately after mixing. Changes of reaction system within 2 min were recorded. The activity of DHAR was expressed as U g^−1^ FW, where U was defined as an increase of 0.01 in absorbance per minute at 290 nm.

Glutathione reductase activity was measured according to the method reported by Halliwell et al. [27]. The reaction system was 3 mL 100 mM phosphate buffer, 0.1 mL 5 mM oxidized glutathione (GSSG), 30 μL 3 mM NADPH, and 0.2 mL crude enzyme solution. NADPH was added to initiate the enzymatic reaction. The absorbance was measured at 340 nm for 2 min. The activity of GR was expressed as U g^−1^ FW, where U was defined as an increase of 0.01 in absorbance per minute at 340 nm.

### 2.11. Determination of AsA-GSH Cycle Substrates and Products Contents

Ascorbic acid and dehydroascorbate content were determined using a kit (Suzhou Keming Biotechnology Co., Ltd., Suzhou, China). AsA was oxidized by ascorbatease AAO to generate DHA, and the AsA content was calculated by measuring the oxidation rate of AsA. AsA content was expressed as nmol/g FW. DHA can be reduced to AsA by DTT, so DHA content can be calculated by measuring AsA formation rate in the system. DHA content was expressed as nmoL/g FW. Determination of GSSG and GSH content was conducted using a kit (Suzhou Keming Biotechnology Co., Ltd.). DTNB reacts with GSH to form a complex with characteristic absorption peak at 412 nm, the absorbance is proportional to GSH content. GSSG and GSH of content were expressed as nmol/g FW.

### 2.12. Statistical Analysis

Data were expressed as means ± standard errors. Figures were prepared with software Origin 8.0 (Northampton, MA, USA). Differences between treatments were analyzed using an ANOVA, followed by LSD multiple range tests at a 0.05 significance level using SPSS 19.0 (SPSS Inc., Chicago, IL, USA).

## 3. Results

### 3.1. Effect of Inoculation of T. roseum at Different pH Conditions on Cell Membrane Permeability and MDA Content of Colonized Apple Tissue

**Figure 1:**
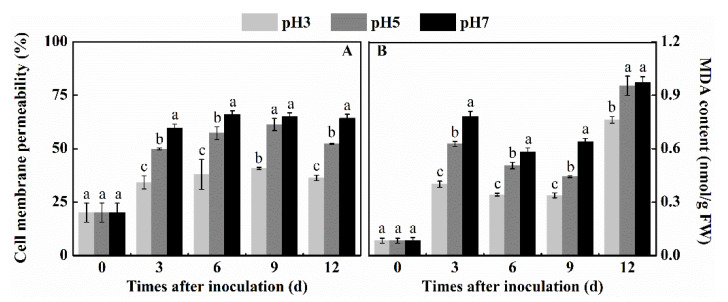
Effect of inoculation of *T. roseum* at different pH conditions on cell membrane permeability (**A**) and malondialdehyde content (**B**) of colonized apple fruits. Bars indicate standard error (±SE). Different letters indicate statistically significant differences (*p* < 0.05) among treatments.

Cell membrane permeability and MDA content are important indexes reflecting the integrity of the cell membranes of the colonized tissue. Cell membrane permeability of fruits inoculated with spore suspensions at different pH levels, showed increasing cell permeability at increasing pH levels. Cell membrane permeability of pH 7 treatment showed the highest activity compared to pH 5 and pH 3 by 23.2% and 77% by the 12th day, respectively (*p* < 0.05) (Figure 1A). In addition, the content of MDA increased as the environmental pH values increased from pH 3 to pH 7. Environmental pH of 7 led to a significantly higher accumulation of MDA than treatments with pH 5 and pH 3 during the first 9 days after inoculation. By the 9th day after inoculation, MDA levels at pH 7 showed values 44% and 89% higher than the values found at pH 5 and pH 3, respectively (*p* < 0.05) (Figure 1B). These results suggest that at increasing environmental pH conditions of colonization by *T. roseum*, the fungus induces enhanced host permeability and MDA accumulation that may contribute to the enhanced colonization at higher pH values.

### 3.2. Effect of Inoculation of T. roseum at Different pH Conditions on NADPH Oxidase and SOD Activities of Colonized Apple Tissue

**Figure 2:**
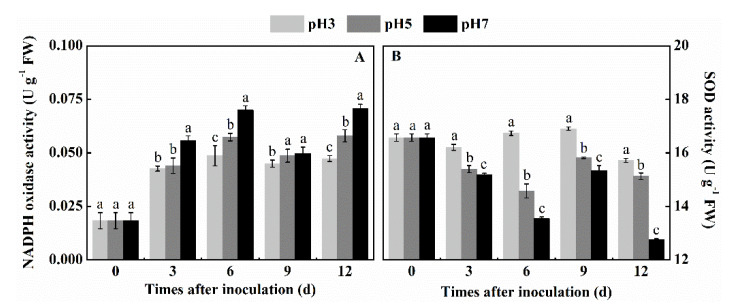
Effect of inoculation of *T. roseum* at different pH conditions on the activities of NADPH oxidase (**A**) and SOD (**B**) of colonized apple fruits. Bars indicate standard error (±SE). Different letters indicate statistically significant differences (*p* < 0.05) among treatments.

NADPH oxidase and SOD are two important enzymes which produce O_2_^●−^ and H_2_O_2_, respectively. Colonized fruits at increasing environmental pH conditions showed increasing NADPH oxidase activities. NADPH oxidase activity showed a positive regulation with the increasing of pH. At the environmental pH 7 it showed 23.8% and 49.3% higher levels than at pH 5 and pH 3 by the 12th day after inoculation, respectively (*p* < 0.05) (Figure 2A). In contrast to NADPH Oxidases, the SOD activity showed a negative regulation of the activity with the increasing of environmental pH. While the SOD activity at pH 3 was not affected in the days after inoculation; exposure of the pathogen to the environmental pH 7 showed a continuous decrease with the increasing days after inoculation (*p* < 0.05) (Figure 2B). The above results indicated that environmental pH increasing at the wound inoculation spot enhanced NADPH oxidase but inhibited SOD activity in apple fruits.

### 3.3. Effect of Inoculation of T. roseum at Different pH Spore Suspensions on the Rate of O_2_^●−^ Production and H_2_O_2_ of Colonized Apple Tissue

**Figure 3:**
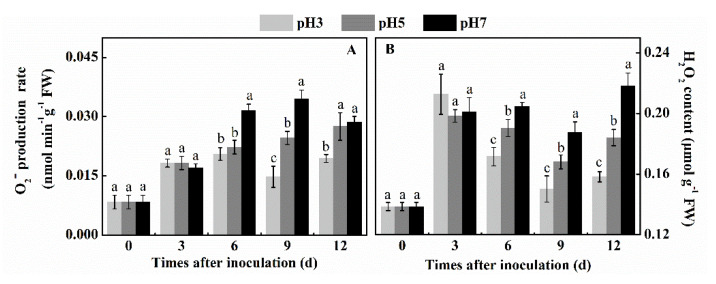
Effect of inoculation of *T. roseum* at different pH conditions on the O_2_^●−^ production rate (**A**), and H_2_O_2_ content (**B**) of colonized apple fruits. Bars indicate standard error (±SE). Different letters indicate statistically significant differences (*p* < 0.05) among treatments.

O_2_^●−^ production rate and H_2_O_2_ content directly reflect the accumulation level of ROS. O_2_^●−^ production rate of fruits inoculated with spores at three pH suspensions showed an overall increase. At pH 7 there was an overall higher O_2_^●−^ production than pH 5 and pH 3. At the 9th day after inoculation, the increase in O_2_^●−^ production rate was 40% and 30% higher than that of pH 5 and pH 3, respectively (*p* < 0.05) (Figure 3A). The H_2_O_2_ accumulation showed a different pattern. While the H_2_O_2_ content of pH 7 treatment increased within 3 days as a result of the inoculation with the pH 7 treatment, it maintained the level of H_2_O_2_ production during all the 12 days of the evaluation. In contrast, the inoculation of spores and environmental pH 3 showed only a single peak of response, 3 days after inoculation followed by a significant decline response up to day 9–12 after inoculation suggesting that there is a different pattern of response between pH 3 and pH 7. Inoculation at pH 5 also showed a similar decline in response to the treatment as the pH 3, however, with an intermediate response between the pH 3 and pH 7 treatment (*p* < 0.05) (Figure 3B). The above results showed that inoculation at increasing pH increased the O_2_^●−^ production rate. However, the pH effect on the H_2_O_2_ content was more complex, showing different responses between pH 3 and pH 7.

### 3.4. Effect of Inoculation of T. roseum at Different pH Conditions on the Activities of CAT and POD of Colonized Apple Tissue

**Figure 4:**
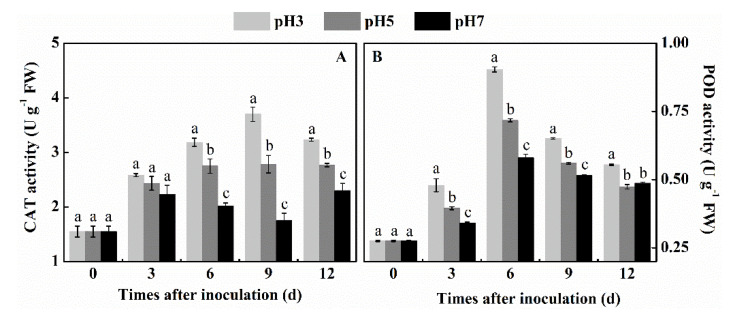
Effect of inoculation of *T. roseum* at different pH conditions on the activity of CAT (**A**) and POD (**B**) of colonized apple fruits. Bars indicate standard error (±SE). Different letters indicate statistically significant differences (*p* < 0.05) among treatments.

CAT and POD are the two key enzymes for H_2_O_2_ scavenging. CAT activity of fruit inoculated with three pH spore suspensions showed an overall increase in activity, but the trend was different between pH 3 and pH 7. With the pH 3 treatment there was a 2-fold increase in enzyme activity by the 3rd day after inoculation, and a 4-fold increase by the 9th and 12th day after inoculation. The fruit response to the inoculation at pH 7 was significantly minor, showing by the 9th day almost no difference of response with the untreated control tissue. At pH 5 an intermediate response with a similar pattern of activity as at pH 3 was observed, suggesting that as the pH increased the level of response of CAT significantly declined (*p* < 0.05) (Figure 4A). POD, an antioxidant that is an endogenous ROS scavenger, again showed the highest increase when spores were inoculated at pH 3 with a 3-fold increase by the 6th day after inoculation and a minimal response when spores were inoculated at pH 7. Interestingly, no response at all was observed by the 3rd day of inoculation, with a slight increase on days 6, 9, and 12 after inoculation (*p* < 0.05) (Figure 4B). The above results indicate a higher level of antioxidant response at lower pH levels and may suggest the importance of the pH increase during fruit colonization.

### 3.5. Effect of Inoculation of T. roseum at Different pH Conditions on the Activities of APX, MDHAR, DHAR, and GR of Colonized Apple Tissue

**Figure 5:**
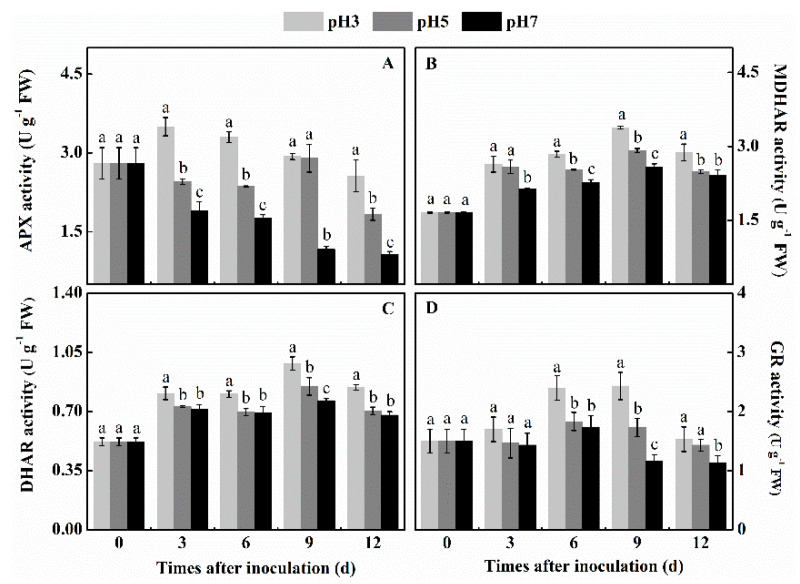
Effect of inoculation of *T. roseum* at different pH conditions on the activity of APX (**A**), MDHAR (**B**), DHAR (**C**), and GR (**D**) of colonized apple fruits. Bars indicate standard error (±SE). Different letters indicate statistically significant differences (*p* < 0.05) among treatments.

APX, MDHAR, DHAR, and GR are involved in the AsA-GSH cycle and play an important role in maintaining AsA and GSH levels as regulators of the redox balance in plants. APX, MDHAR, DHAR, and GR are also H_2_O_2-_scavenging enzymes and are indispensable for the protection of chloroplasts and other cell constituents from damage by H_2_O_2_ in fruits. The inoculation of spores at pH 3 in all the 4 enzymes analyzed showed a significantly increased response during the 12 days of evaluation. For MDHAR and DHAR at pH 7 the level of increase of activity was 20%–30% lower than for pH 3 (*p* < 0.05) (Figure 5B,C). However, for APX and GR activities, the level of activities were even inhibited compared to those at pH 3 (Figure 5A,D). This clearly indicates that while the antioxidant activity pattern at pH 3 is induced, the response at pH 7 is significantly inhibited or not activated.

### 3.6. Effect of Inoculation of T. roseum at Different pH Conditions on the Contents of AsA, DHA, GSH, and GSSG of Colonized Apple Tissue

**Figure 6:**
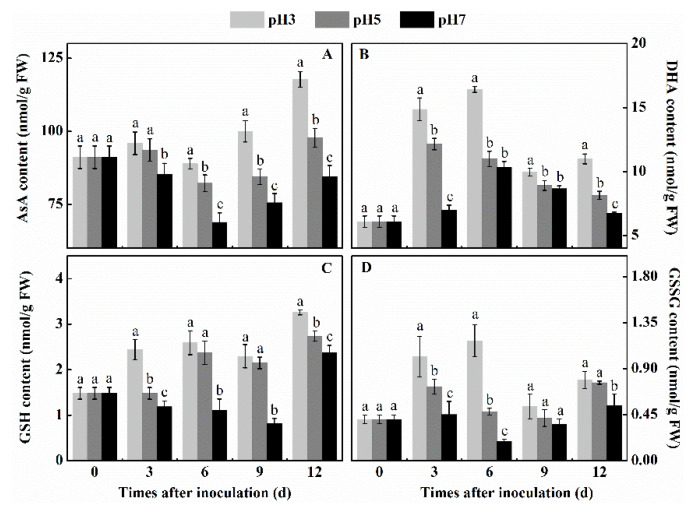
Effect of inoculation of *T. roseum* at different pH conditions on the activity of AsA (**A**), DHA (**B**), GSH (**C**), and GSSG (**D**) of colonized apple fruits. Bars indicate standard error (±SE). Different letters indicate statistically significant differences (*p* < 0.05) among treatments.

AsA and GSH are the main antioxidant substances in the ASA-GSH cycle, which can scavenge H_2_O_2_ through the ASA-GSH cycle. The general pattern of response of the 4 antioxidants showed a negative response as the pH increased (Figure 6). While there was a differential increase in the level of antioxidants, there was a clear and significant decrease at pH 7 compared to the colonization at pH 3. Analysis of each of the antioxidants showed two different responses: two of these antioxidants ASA and GSH (Figure 6A,C) showed patterns of continuous (in general) increase in concentrations when the inoculation was carried out at pH 3 at different periods after inoculation, but the other two, GSH and GSSG (Figure 6B,D) showed only an initial positive response after inoculation (3 and 6 days) and then a decline in the concentrations (9 and 12 days) at similar time levels as at pH 7. These data indicate that the activation of the antioxidant content is strongly dependent on the pH, being higher at low pH values, but still showing differential levels of accumulation according to the specific antioxidant.

## 4. Discussion

Postharvest pathogens have the ability to alkalinize the environment, contributing to fungal pathogenesis by activation of genes that modulate the pathogenicity factors [28]. Inoculation of apple fruits with spore suspensions of *T. roseum* at increasing pH values, from pH 3 to pH 7, enhanced a faster colonization of the apple tissue and the enhancement of secretion of pectolytic enzymes, as a result of the enhanced pH induced by the pathogen [7]. However, the increase of pH modulated host physiological responses in the apple tissue. Inoculation of spores of the pathogen at pH 7 induced a significant increase in NADPH oxidase activity, a faster rate of increase of O_2_ production, the highest H_2_O_2_ accumulation, and reduction of cell membrane integrity. A similar pattern of responses was observed for the secretion of ammonia by *C. coccodes*, which activated the tomato fruit RBOH activity, thus regulating the host H_2_O_2_ level [16]. Given that ammonia can activate H^+^-ATPase activity, which affects the influx of Ca^2+^ [14], it is possible that Ca^2+^ can activate calcium-dependent protein kinases (CDPKs) at downstream targets [29] and then generate of O_2_^●−^ by phosphorylating NADPH oxidase [30]. Therefore, fungal alkalization of the environment might activate the cell membrane proton pump H^+^-ATPase under the higher pH 7 environment, leading to Ca^2+^ influx, which could further activate CDPK, resulting in an accumulation of ROS. The ROS could directly attack the membrane system, thus destroying the integrity of the cell membrane, and contribute to the enhanced fungal colonization. However, this still requires further study to fully elucidate this mechanism.

ROS is an important virulence factor of pathogens, which can react with lipids, proteins, nucleic acids, and other biological macromolecules of host cells, leading to lipid peroxidation, protein cross-linking, DNA damage, and so forth, finally resulting in the programmed death of host cells [31]. However, other antioxidant systems regulate the accumulation of ROS in the fruit tissue. The present observations indicated that the SOD, CAT, and POD activities were strongest in pH 3 inoculated fruits and weakest in pH 7 inoculated fruits. These activities are important given that SOD is the only enzyme that can interact with O_2_^●−^ to generate H_2_O_2_ in organisms [32]. CAT is also one of the key enzymes in the antioxidant system of plants, and its function is to scavenge H_2_O_2_, which can reduce H_2_O_2_ to H_2_O and O_2_ [33] showing a closely related activity to the redox balance in plant cells [34]. POD is considered an endogenous ROS scavenger in plant cells, working in concert with CAT to form the antioxidant enzyme system of plants [35,36]. Previous reports indicated that transcript analysis of SOD, CAT, and POD in petunia showed significantly higher activities at ambient pH 3 than pH 5.8 [37]. Similarly, higher activities of both SOD and CAT were observed at pH 3 than pH 7 in *Rosa hybrida* (cv. Tereasa) [38], supporting the present study. Therefore, it is clear that lower pH values were more conducive to maintaining the activity of SOD, CAT, and POD than higher pH environments. However, another antioxidative system was also significantly activated at lower pH. The AsA-GSH cycle is an important H_2_O_2_ scavenging system in plants, which plays an indispensable role in the effective removal of H_2_O_2_ [39]. Our results indicate that in apples colonized by *T. roseum* APX, MDHAR, DHAR, and GR activities, as well as the levels of AsA, DHA, GSH, and GSSG at pH 3, were significantly higher than at pH 5 and pH 7. APX is the first enzyme in the cycle that specifically catalyzes the reaction of AsA with H_2_O_2_ to form MDHA [40]. Results obtained in colonized tissue with *Trichotecium* were similar to those found in ginger seedlings that showed strong activity of the AsA-GSH cycle enzymes under low pH conditions [12]. Yin et al. found that low pH improved APX activity and increased glutathione and ascorbic acid content, thus effectively scavenging H_2_O_2_ and protecting the integrity of membrane lipids [12]. In fruit systems such as kiwi fruit treated with 5 mM oxalic acid (pH 3.1), the low pH increased the AsA content, thereby enhancing the ROS scavenging ability and reducing the damage of membrane lipid peroxidation [41]. Therefore, we speculated that low pH conditions could increase the activity of the AsA-GSH cycle, and thus effectively scavenge excessive H_2_O_2_. In summary, we hypothesize that the colonization by *T. roseum* of apple fruit, under reduced pH conditions, induced the activation of the AsA-GSH cycle, contributing to the reduced colonization of the fungus in apple fruit.

## 5. Conclusions

Apple fruits colonized by *T. roseum* spore suspensions at different pH values modulated the activity of pectolytic and cellulolytic enzymes at increasing pH values, but the local environmental pH levels have differential effects on ROS production, cell membrane integrity, and ROS scavenging systems. Fruits colonized at pH 3 showed the lowest rate of O_2_^●−^ production and H_2_O_2_ accumulation, the highest cell membrane integrity, and SOD, CAT, and POD activities, and the strongest AsA-GSH cycle activity. However, the metabolic responses at the highest pH tested were the opposite. These results suggest that the metabolic response of the fruit resulting from the alkalization by the secretion of ammonia is a key factor for modulation of the colonization of the fruit by *Trichotecium*. The present results indicate that fungal pH modulation of the environment may modulate fungal metabolic processes as well as fruit metabolic processes, both of them contributing to the modulation of fungal colonization by the pathogen in ripening fruits [42].

## Data Availability

Data available in a publicly accessible repository.
